# New Markers of Inflammation and Tubular Damage in Children with Chronic Kidney Disease

**DOI:** 10.1155/2017/9389432

**Published:** 2017-07-20

**Authors:** Kinga Musiał, Agnieszka Bargenda, Dorota Drożdż, Danuta Zwolińska

**Affiliations:** ^1^Department of Pediatric Nephrology, Wrocław Medical University, Wrocław, Poland; ^2^Department of Pediatric Nephrology, Jagiellonian University, Kraków, Poland

## Abstract

**Introduction and Aims:**

Monocyte chemoattractant protein- (MCP-) 1, macrophage colony-stimulating factor (MCSF), and neopterin are connected with monocyte migration and transition into macrophages, leading to fibrosis and tubular damage in the course of CKD. The aim of the study was to analyze the applicability of urinary fractional excretion (FE) of MCP1, MCSF, and neopterin, as markers of inflammation and tubular damage, in children with CKD.

**Methods:**

The study group consisted of 61 children with CKD stages 1–5 and 23 age-matched controls. The serum and urine concentrations of MCP1, MCSF, and neopterin were assessed by ELISA and then the fractional excretion (FE) was calculated.

**Results:**

FE MCSF and neopterin values exceeded 1% already in controls. FE MCSF rose significantly since CKD stages 1-2, FE neopterin since CKD stages 3–5. FE MCP1 was below 1% in healthy controls and in CKD stages 1-2, then increased significantly in CKD stages 3–5.

**Conclusions:**

The FE MCP-1 values show that inflammation precedes the tubular dysfunction. FE MCSF and FE neopterin may be considered new markers of the renal parenchyma progressive damage. Fractional excretion may become a useful tool in the assessment of inflammation and tubular damage in children with CKD.

## 1. Introduction

Chronic kidney disease (CKD) is characterized by enhanced migration of immunocompetent cells to the sites of inflammation and subsequent renal fibrosis [1]. The latter is an essential element of progressive and irreversible tubular damage [2]. Various chemotactic agents influence the abovementioned CKD-related complications.

Monocyte chemoattractant protein- (MCP-) 1 and macrophage colony-stimulating factor (MCSF) control early stages of cell migratory activity, such as movement of monocytes to the sites of inflammation and their transition into macrophages [3]. Thus, the activity of MCP-1 and MCSF may be treated as a substitute of the inflammation process intensity and monocyte and macrophage activity, as well as the cell damage in the course of chemotactic migration and transition. Animal studies have shown profibrotic activity of MCP-1 and its localization in tubular cells [4, 5]. The MCP-1 gene (MCP-1-2518 A/G) polymorphism has been analyzed in adults on hemodialysis and in children with focal segmental glomerulosclerosis [6, 7]. MCSF was assessed in adults on hemodialysis [8]. However, MCP-1 or MCSF has never been put into the focus of chronic kidney disease progression in children.

Neopterin was the only exception in this group, since its production is not a cause, but a consequence of enhanced cell migration and ongoing inflammation. In detail, both monocytes and macrophages excrete neopterin upon stimulation. Therefore, this molecule may be a marker of cellular immune response. The increased serum and urine concentrations of neopterin were observed in adults with nephrotic syndrome, with advanced stages of CKD, and on hemodialysis [9, 10]. The elevated excretion of neopterin with urine was also noticed in adults with mesangial proliferative glomerulonephritis [11]. Neopterin was also useful as a risk marker of renal allograft rejection [12].

However, the abovementioned parameters have never been analyzed as markers of inflammation or macrophage activity in children with CKD.

In clinical practice, the analysis of fractional excretion (FE) as a substitute of tubular dysfunction in the CKD patients was restricted to phosphate metabolism so far [13]. Scientific approach to FE concerned heat shock protein (Hsp27) in adults [14] and markers of apoptosis in children from our previous study [15]. That investigation confirmed the usefulness of FE in depicting tubular damage and epithelial-mesenchymal transition in the patients with CKD. However, neither MCP-1, MCSF, nor neopterin has been tested, in the light of fractional excretion, as potential markers of tubular damage in the course of chronic kidney disease.

Therefore, the aim of this study was to analyze the factors engaged in monocyte activation, migration, and their transition into macrophages, by assessing the concentrations of MCP-1, MCSF, and neopterin, in the serum and urine of children with CKD and of controls. We also evaluated the usefulness of fractional excretion (FE) of MCP-1, MCSF, and neopterin as pluripotent markers of inflammation, monocyte-macrophage interplay, and tubular damage in the course of CKD.

## 2. Methods

### 2.1. Patient Characteristics

Eighty-four patients enrolled in this study were divided into 3 groups. The first group (CKD I) contained 20 children with CKD stages 1-2, and the second group (CKD II) consisted of 41 patients with CKD stages 3–5. Twenty-three children with primary nocturnal enuresis and normal kidney function served as controls.

Basic clinical data are shown in Table 1.

The diseases leading to CKD were reflux nephropathy (19 cases), obstructive uropathy (14 patients), hypo-/dysplastic kidneys (12), chronic glomerulonephritis (10), polycystic kidney disease (4), and hemolytic uremic syndrome (2).

None of the patients has shown clinical evidence of infection; had diabetes, malignancies, or vasculitides; has smoked; and took antibiotics and statins. The patients were also free of such comorbidities like cardiovascular disease, peripheral vascular disease, or obesity. In the CKD group, 35 children were normotensive according to the criteria of the European Society of Hypertension in children and adolescents [16]. In 16 patients, blood pressure was well controlled with the use of ACE inhibitors (8 children), calcium channel blockers (6 patients), and *β*-blockers (2 children), and 10 patients needed combined therapy. In all patients at CKD stages 3–5, phosphate binders and vitamin D metabolites were supplemented.

Informed consent was obtained from the subjects and their parents, if necessary. The research project has been approved by the university ethics committee, in accordance with the Helsinki declaration.

Blood samples were drawn from the peripheral veins after an overnight fast. Samples were clotted for 30 minutes and centrifuged for 10 minutes, and then serum was stored at −20°C until assayed. Urine was collected aseptically from the first morning sample, centrifuged for 10 minutes, and then stored at −20°C until assayed.

### 2.2. Assay Characteristics

The serum and urine concentrations of MCP-1, MCSF, and neopterin were evaluated by commercially available ELISA kits (MCP-1—R&D Systems, reagent kit DCP00; MCSF—R&D Systems, reagent kit DMC00B; neopterin—IBL International, reagent kit RE59321). Standard, serum, and urine samples were transferred to 96-well microplates precoated with recombinant antibodies to human MCP-1, MCSF, and neopterin. Measurements were performed according to the manufacturer's instructions, and results were calculated by reference-to-standard curves.

The serum and urine creatinine were assessed with the Creatinine OSR61204 reagent on the Beckman Coulter AU2700 analyzer.

The fractional parameter excretion was calculated according to the formula ([urine parameter concentration] × [serum creatinine concentration]) / ([serum parameter concentration] × [urine creatinine concentration]) × 100%.

### 2.3. Statistical Analysis

The results are expressed as median values and interquartile ranges. Since the null hypothesis of normality of distribution was rejected by the Shapiro-Wilk test, comparisons were evaluated by using nonparametric tests (Kruskal-Wallis, Mann–Whitney *U*). Relations between parameters were defined by Spearman's correlation coefficient *R*. Statistical analysis was performed using the package Statistica ver. 12.0 (StatSoft). A *p* value < 0.05 was considered significant.

## 3. Results

### 3.1. Serum and Urine Concentrations of MCP-1, MCSF, and Neopterin

The serum and urine concentrations of all examined parameters were significantly elevated, irrespective of the CKD stage, versus controls (Tables 2 and 3). Serum MCSF levels rose gradually with the CKD progression, whereas serum MCP-1 and neopterin concentrations in advanced stages of CKD were significantly lower than in mild CKD, although still above the values seen in the control group (Table 2).

Urine MCP-1 levels were significantly increased in CKD stages 1-2 versus controls, but then remained stable despite progression of CKD to stages 3–5 (Table 3). Urine MCSF concentrations decreased in advanced versus mild CKD. Urine neopterin levels were the only ones increasing gradually with the CKD progression (Table 3).

Interestingly, the values of MCSF and neopterin in urine were always higher than the relevant ones in serum, irrespective of the analyzed group.

### 3.2. Fractional Excretion of MCP-1, MCSF, and Neopterin

The fractional excretion (FE) of MCP-1 did not exceed 1% in healthy controls, whereas it reached up to 5% in the case of MCSF and neopterin (Table 4). Only MCP-1 and MCSF FE values were significantly elevated in children with CKD stages 1-2 versus controls, whereas all FE values were higher in patients with CKD stages 3–5 versus those with CKD stages 1-2 (Table 4). However, they remained below 1% for MCP-1 in children with CKD stages 1-2. The fractional excretion of MCSF and neopterin was far beyond 1% in all CKD patients.

### 3.3. Correlations

The MCP-1, MCSF, and neopterin serum levels correlated significantly with each other. In detail, MCP-1 correlated positively with neopterin (*R* = 0.77; *p* < 0.00001) and negatively with MCSF (*R* = −0.55; *p* < 0.000001). Neopterin correlated negatively with MCSF (*R* = −0.66; *p* < 0.000001). There was no correlation between serum and urine values, although in the case of MCP-1, the statistical significance was borderline (*p* = 0.057). The urine concentrations of analyzed parameters did not correlate with each other. None of the parameters correlated with eGFR.

## 4. Discussion

Our investigation has revealed elevated concentrations of MCP-1, MCSF, and neopterin in the serum and urine of children with chronic kidney disease in comparison to those in the control group. However, the dynamics of these changes differed between the analyzed molecules.

The MCP-1 concentrations in serum and urine have risen significantly already in CKD stages 1-2. There are no relevant data on MCP-1 levels in patients with CKD treated conservatively; thus, the interpretation of these results seemed challenging. Since none of the parameters has correlated with eGFR, the source of serum elevation was rather overproduction than accumulation, whereas increased excretion with urine showed indirectly the monocyte activation in renal interstitium. This might mean that MCP-1 is a useful marker of inflammation, and monocyte activity in specific, both in serum and in urine. Moreover, since kidney MCP-1 is mainly localized in the proximal tubules, urine MCP-1 concentrations may mirror their function.

Of note, the fractional excretion, never tested in the case of MCP-1, might be of an added value in the assessment of tubular status quo. Indeed, the values of FE MCP-1 in CKD stages 1-2, significantly higher than in controls, but not exceeding 1%, seemed to confirm the early inflammatory process in the tubules, preceding their damage. The latter has become evident in CKD stages 3–5, when FE values kept rising and exceeded 1%. Thus, fractional excretion could be a useful tool in distinguishing between the inflammatory overactivity and irreversible tubule damage, resulting in excessive excretion of MCP-1 with urine.

The role of FE MCP-1 in the assessment of tubular damage might be strengthened by the fact that, in children with CKD stages 3–5, the MCP-1 serum concentration declined, whereas the urinary one remained stable, in comparison to patients with CKD stages 1-2. Therefore, monocyte activity seemed either to diminish (as in serum) or to become stable (as in urine) in the course of advanced CKD. Hence, FE MCP-1 rising values have shown the switch from the inflammatory profile in early CKD to the tubular dysfunction in late stages of the disease.

MCSF is another inflammation marker connected with macrophage activity and migration. Similar to MCP-1, both serum and urine concentrations rose in the early stages of CKD, independent of eGFR values, and serum concentrations did not correlate with the urinary ones. Like in the case of MCP-1, no comparative data from other studies exist. However, such increment in serum could be interpreted as a result of macrophage activation and inflammation rather than accumulation of the molecule, whereas the rise in urine might be the result of MCSF generation by renal parenchyma. Contrary to MCP-1, the serum MCSF values continued to rise with CKD progression, whereas the urine concentrations declined. Yet, the values of MCSF in urine outreached those in serum, in controls as well as in patients, irrespective of the CKD stage. Thus, probably MCSF has shown more propensity towards the activity of macrophages in serum than of those in renal interstitium. The decline of MCSF urine concentrations could also mirror the progression of renal fibrosis, which is macrophage-mediated in its early stages, but then becomes independent of cellular activity and irreversible.

Some of those uncertainties have clarified when the data on fractional excretion were taken into account. The FE MCSF values exceeded 1% already in controls and kept rising with the progression of CKD. Therefore, MSCF presence in urine most probably resulted from its renal production, which seems a constant feature, independent of renal function. Consequently, the FE MCSF values, aggravating with renal failure progression, could signify early inflammatory overactivity in renal parenchyma of CKD stages 1-2 and then renal fibrosis and tubular damage in CKD stages 3–5.

Neopterin was the last analyzed parameter connected with monocyte-macrophage activity and inflammation. The increased molecule concentrations in serum and urine, seen in all CKD children, were concordant with previous observations concerning adults with advanced CKD [9]. Yadav et al. [10] have noticed the serum concentrations of neopterin, increasing with declining eGFR in adults with CKD stages 3–5, and on hemodialysis. However, the comparison of those results with ours seems difficult, since the adult population, with diabetic and hypertensive nephropathy responsible for additional inflammatory triggers, differs from children with congenital anomalies in the urinary tract as a main cause of CKD. Meanwhile, our observation was the first one in the pediatric population and in the early stages of CKD.

Like in the case of MCSF, the neopterin values in urine were higher than the ones in serum, even in the control group, and did not correlate with each other. The serum neopterin concentrations in CKD stages 3–5 diminished versus those in CKD stages 1-2, mimicking the activity of MCP-1. Thus, neopterin in serum seemed dedicated to monocyte rather than macrophage activity. In the meantime, neopterin has shown the unique systematic increment of the urine values, with significant differences between early and late stages of CKD. This would make urinary neopterin a good candidate marker of late changes in renal parenchyma, like persistent inflammation facilitating progression of renal failure [1].

The FE values of neopterin seemed to confirm this hypothesis, since they remained unchanged in CKD stages 1-2 versus controls and then rose significantly in CKD stages 3–5. Moreover, they were above 1%, both in controls and in CKD children, suggesting neopterin is produced by the healthy renal parenchyma and, to a greater extent, by the damaged tissue. Thus, FE neopterin values in advanced CKD could mirror the extent of tubular damage due to persistent inflammation.

Taken together, the complex analysis of serum, urine, and fractional excretion values of examined parameters enabled differentiation between early and persistent inflammation, as well as analysis of the progression of tubular damage.

## 5. Conclusions

Fractional excretion of the examined markers may become a useful tool in the assessment of inflammation and tubular damage in the course of chronic kidney disease. The FE MCP-1 values show that the inflammatory process precedes the tubular dysfunction. FE MCSF and FE neopterin may be considered new markers of renal parenchyma progressive damage in children with CKD.

## Figures and Tables

**Table 1:** Basic characteristics of the patients.

Parameters	Median (lower-upper quartile)		
	Control group (*n* = 23)	CKD I (*n* = 20)	CKD II (*n* = 41)
Age (years)	10.5 (5.0–16.5)	9.5 (4.0–12.5)	11.0 (4.5–16.5)
Gender	13 girls	5 girls	17 girls
	10 boys	15 boys	24 boys
eGFR (ml/min/1.73 m^2^)	97.1 (92.3–115.0)	79.5 (65.7–97.4)	26.2 (17.3–41.5)

**Table 2:** Serum concentrations of examined parameters in CKD children and in the control group.

Parameters in serum	Median value (lower-upper quartile)		
	Control group (*n* = 23)	CKD I (*n* = 20)	CKD II (*n* = 41)
sMCP-1 (ng/ml)	348.3 (328.9–432.2)	1157.6^a^ (1143.2–1183.7)	994.3^b^ (977.2–1013.0)
sMCSF (pg/ml)	258.2 (253.7–265.0)	874.2^a^ (864.9–895.4)	989.2^b^ (965.4–1031.3)
s neopterin (ng/ml)	23.3 (22.8–23.5)	92.9^a^ (91.9–95.5)	84.5^b^ (82.4–88.0)

Mann–Whitney *U* test: ^a^*p* < 0.0001, CKD I versus the control group. ^b^*p* < 0.001, CKD II versus CKD I.

**Table 3:** Urine concentrations of examined parameters in CKD children and in the control group.

Parameters in urine	Median value (lower-upper quartile)		
	Control group (*n* = 23)	CKD I (*n* = 20)	CKD II (*n* = 41)
uMCP-1 (ng/mg creatinine)	88.4 (78.5–101.2)	872.5^a^ (798.4–940.2)	804.4 (658.4–924.8)
uMCSF (pg/mg creatinine)	1004.4 (963.7–1125.2)	6948.4^a^ (6296.9–7566.2)	5940.8^b^ (5441.8–7464.7)
u neopterin (ng/mg creatinine)	112.31 (101.1–131.6)	300.1^a^ (282.1–324.3)	501.76^b^ (446.2–667.0)

Mann–Whitney *U* test: ^a^*p* < 0.0001, CKD I versus the control group. ^b^*p* < 0.01, CKD II versus CKD I.

**Table 4:** Fractional excretion of examined parameters in CKD children and in the control group.

Fractional excretion (FE) of parameters	Median value (lower-upper quartile)		
	Control group (*n* = 23)	CKD I (*n* = 20)	CKD II (*n* = 41)
FE MCP-1 (%)	0.18 (0.14–0.19)	0.42^a^ (0.36–0.47)	1.58^b^ (0.89–3.02)
FE MCSF (%)	2.79 (2.63–3.06)	4.40^a^ (3.93–4.93)	11.67^b^ (6.72–21.18)
FE neopterin (%)	4.53 (3.64–5.32)	5.96 (5.36–6.63)	6.49^b^ (4.21–11.80)

Mann–Whitney *U* test: ^a^*p* < 0.0001, CKD I versus control group. ^b^*p* < 0.00001, CKD II versus CKD I.
